# *Bacteroides fragilis* Prevents *Clostridium difficile* Infection in a Mouse Model by Restoring Gut Barrier and Microbiome Regulation

**DOI:** 10.3389/fmicb.2018.02976

**Published:** 2018-12-21

**Authors:** Huimin Deng, Siqi Yang, Yucheng Zhang, Kai Qian, Zhaohui Zhang, Yangyang Liu, Ye Wang, Yang Bai, Hongying Fan, Xinmei Zhao, Fachao Zhi

**Affiliations:** ^1^Guangdong Provincial Key Laboratory of Gastroenterology, Department of Gastroenterology, Institute of Gastroenterology of Guangdong Province, Nanfang Hospital, Southern Medical University, Guangzhou, China; ^2^Guangzhou ZhiYi Biotechnology Co., Ltd., Guangzhou, China; ^3^Guangdong Provincial Key Laboratory of Tropical Disease Research, School of Public Health, Southern Medical University, Guangzhou, China

**Keywords:** next-generation probiotic, gut barrier, gut microbiota, *Clostridium difficile*, commensal bacteria

## Abstract

*Clostridium difficile* is currently the leading cause of nosocomial infection. Antibiotics remain the first-line therapy for *C. difficile*-associated diseases (CDAD), despite the risks of resistance promotion and further gut microbiota perturbation. Notably, the abundance of *Bacteroides fragilis* was reported to be significantly decreased in CDAD patients. This study aimed to clarify the prophylactic effects of *B. fragilis* strain ZY-312 in a mouse model of *C. difficile* infection (CDI). The CDI mouse model was successfully created using *C. difficile* strain VPI 10463 spores, as confirmed by lethal diarrhea (12.5% survival rate), serious gut barrier disruption, and microbiota disruption. CDI model mice prophylactically treated with *B. fragilis* exhibited significantly higher survival rates (100% in low dosage group, 87.5% in high dosage group) and improved clinical manifestations. Histopathological analysis of colon and cecum tissue samples revealed an intact gut barrier with strong ZO-1 and Muc-2 expression. The bacterial diversity and relative abundance of gut microbiota were significantly improved. Interestingly, the relative abundance of *Akkermansia muciniphila* was positively correlated with *B. fragilis* treatment. *In vitro* experiments showed that *B. fragilis* inhibited *C. difficile* adherence, and attenuated the decrease in CDI-induced transepithelial electrical resistance, ZO-1 and MUC-2 loss, and apoptosis, suggesting that *B. fragilis* protected against CDI possibly by resisting pathogen colonization and improving gut barrier integrity and functions. In summary, *B. fragilis* exerted protective effects on a CDI mouse model by modulating gut microbiota and alleviating barrier destruction, thereby relieving epithelial stress and pathogenic colitis triggered by *C. difficile*. This study provides an alternative preventative measure for CDI and lays the foundations for further investigations of the relationships among opportunistic pathogens, commensal microbiota, and the gut barrier.

## Introduction

The overuse of antibiotics is currently regarded as the most common reason for disturbance of gut microbiota, and if the disruption reaches a certain level, the host can develop *Clostridium difficile*-associated disease (CDAD) (He et al., 2013; Tang et al., 2016). *C. difficile*, the leading cause of AAD, is a Gram-positive, spore-forming, opportunistic pathogenic anaerobe. As the major culprit for nosocomial infections, it has captured wide public attention for its high morbidity and mortality rates (Kelly and LaMont, 2008; He et al., 2013; Lessa et al., 2015). CDAD is characterized by a spectrum of diseases ranging from clinical diarrhea to pseudomembranous colitis and toxic megacolon. The *in vivo* pathogenesis of *C. difficile* is mainly dependent on two macromolecular toxins, toxin A and toxin B. These toxins target and destroy intestinal epithelial cells by modifying cytoskeletal components and disrupting tight cellular junctions, ultimately leading to apoptosis and disruption of gut barrier integrity (Pothoulakis, 2000; Rineh et al., 2014).

The first-line therapy for *C. difficile* infection (CDI) remains antibiotics, mainly MTZ, vancomycin, and fidaxomicin (Mushtaq, 2018). Although antibiotics are regarded as the most efficient therapy in clinical practice, the risks of resistance promotion and further gut microbiota perturbation cannot be ignored. Therefore, effective alternative non-antibiotic therapies are urgently needed and have been researched for several years. Recently, bacteriotherapy has gradually become accepted as a solution for CDI treatment (Lawley et al., 2012; van Nood et al., 2013; Shen et al., 2017; Mills et al., 2018). An integrated gut barrier and a healthy microbiota net are vital to resist *C. difficile*. Therefore, complementing bacteria in a timely manner to rebuild a health-associated microbiota net seems more reasonable. Recent clinical studies have proven that FMT has excellent therapeutic effects in CDI patients (van Nood et al., 2013; Kelly and Tebas, 2018). However, despite the high clinical remission rate, FMT therapy still has safety concerns including the risk of unexpected infections by undetected pathogenic bacteria. Moreover, the exact mechanisms of FMT therapy for CDI remission remain to be clearly elucidated.

*Bacteroides fragilis* (*B. fragilis*) was reported to have an inverse association with *C. difficile* (Goldberg et al., 2014) in a study of 59 patients including CDI or non-CDI patients, indicating that *B. fragilis* probably plays a role in the protection from CDI. We previously demonstrated that *B. fragilis* exerted protective effects in an AAD rat model through microbiome regulation and gut barrier integrity restoration (Zhang et al., 2018). Therefore, in the present study, we examined the protective effects of *B. fragilis* in a mouse model of CDI and explored the possible mechanism involving microbe-epithelium interactions. As we know, an intact gut barrier is vital for natural host defenses against *C. difficile* (Buonomo et al., 2016; Winston and Theriot, 2016; Crobach et al., 2018). Many factors contribute to intestinal barrier function, including epithelial integrity, a thick mucus layer, healthy gut microbiota, and a stable immune system (Bron et al., 2017). Normally, colon epithelial cells are sealed with tight junction proteins to form a strong barrier, while mucins act as an impermeable diffusion barrier that lies over the epithelium. Muc-2 protein is the most common type of mucin expressed in the colon and ZO-1 protein is a representative tight junction protein. In this study, we explored the influence of *B. fragilis* on Muc-2 and ZO-1 expression *in vivo* and *in vitro*.

Our data demonstrated that *B. fragilis* ZY-312 significantly reduced mortality in the CDI mouse model, associated with the restoration of gut barrier integrity and regulation of microbiota. *In vitro* experiments suggested that *B. fragilis* inhibited *C. difficile* adherence to colon cells and reduced *C. difficile*-induced apoptosis, and Muc-2 and ZO-1 loss.

## Materials and Methods

### Ethics Statement

All animal experiments were approved by Nanfang Hospital Animal Ethics Committee (approval number: NFYY-2014-123) and were in accordance with relevant ethical principles and guidelines set by the Animal Welfare Act and the NIH Guide for the Care and Use of Laboratory Animals. Experiments involving isolation of *B. fragilis* strain ZY-312 from infant fecal samples were approved by the Medical Ethics Committee of Nanfang Hospital (approval number: NFEC-2014-040).

### Bacteria Strains, Cell Lines, and Culture Conditions

*Bacteroides fragilis* strain ZY-312 was described in detail in our previous report (Deng et al., 2016; Wang et al., 2017), and the same cultivation conditions were used. *C. difficile* strain VPI 10463 was purchased from the American Type Culture Collection (Manassas, VA, United States) and verified by PCR amplification. Strain VPI 10463 was cultured anaerobically in sterile tubes containing 4 mL of TSB supplemented with 20% FBS at 37°C in an anaerobic glove box (DG250; Don Whitley Scientific, Bingley, United Kingdom) for 24 h.

The human colon carcinoma cell lines HT-29, Caco-2, IEC-6, and Vero were provided by Southern Medical University (Guangzhou, China), and routinely cultured in RPMI 1640 medium (Gibco, Life Technologies, Carlsbad, CA, United States) supplemented with 10% heat-inactivated FBS (PAN, Aidenbach, Germany), penicillin (100 U/ml), and streptomycin (100 ng/ml) in an incubator at 37°C under 95% (v/v) humidified air and 5% (v/v) CO_2_.

### Preparation of *C. difficile* VPI 10463 Spores

Sporulation of VPI 10463 was induced as described previously (Sorg and Sonenshein, 2008). Briefly, strain VPI 10463 was stored at a ratio of 1:1 with glycerin at -80°C before sporulation. For sporulation, 20 μL of the stored solution was seeded in BHI agar (BD Biosciences, San Jose, CA, United States) containing 0.1% taurocholate (Sigma, St. Louis, MO, United States) and incubated anaerobically at 37°C for 7 days. More than 80% of spores in individual microscope fields were considered eligible for subsequent experiments (Supplementary Figure S1A). After incubation, 3 mL of sterilized ice-cold water per agar plate was used to wash out the vegetative cell-spore mixed colonies. The resulting solution was collected in a sterilized tube, centrifuged at 3000 rpm for 5 min, and washed once. The pellet was resuspended in 10 mL of 50% (w/v) sucrose solution and centrifuged at 3000 rpm for 20 min. After five washes with cold water by centrifugation at 3000 rpm for 20 min, the samples were heated to 60°C for 30 min to kill the vegetative bacteria. The concentration of the spore solution was determined by a serial dilution method on BHI agar containing 0.1% taurocholate. The spore solution was stored at -80°C until use. The toxicity of the spores was determined using a C. Diff Quik Chek Complete Detection Kit (Techlab, Blacksburg, VA, United States).

### Animals and CDI Model

**FIGURE 1:**
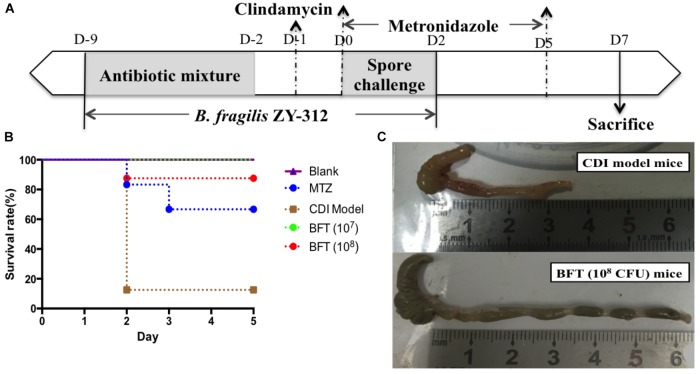
*Bacteroides fragilis* ZY-312 prevents CDI-associated death. **(A)** Experimental design for *B. fragilis* ZY-312 prophylactic treatment of the CDI model mice. The first step of inducing the CDI mouse model was to use a large dosage of antibiotics to disrupt the normal gut microbiota. From D –9 to D –2, drinking water with antibiotics including kanamycin (0.8 mg/mL), gentamicin (0.07 mg/mL), colistin (0.1135 mg/mL), metronidazole (0.43 mg/mL), and vancomycin (0.09 mg/mL) was offered to male C57BL/6 mice (6–8 weeks old, *n* = 6–8). Clindamycin (10 mg/kg) was intraperitoneally injected the day (D –1) after the cessation of drinking water with antibiotics. Then the mice were orally challenge with 3 × 10^8^ cfu of *Clostridium difficile* VPI 10463 spores from D0 to D2. To explore the prophylactic effects of *B. fragilis* on preventing *C. difficile*-associated diseases, 1 × 10^7^ or 1 × 10^8^ cfu/day *B. fragilis* were given, respectively, to CDI model mice from D –9 to D2. Metronidazole (1 mg/day) was given orally from D0 to D5, as the positive control. **(B)** Survival rates of mice in the Blank, MTZ (Metronidazole treated), CDI model, and BFT (*B. fragilis* treatment) groups. **(C)** Visual observations of colon tissues from CDI model mice (top) and BFT (10^8^ cfu) mice (means ± SEM; *n* = 6–8 per group).

To induce the CDI model, 6–8-week-old male C57BL/6 mice were obtained from the Animal Experiment Center of Guangdong Province (China) and reared under specific pathogen-free conditions in the Southern Medical University animal facility. All animals had appropriate space, with free access to tap water and a standard rodent diet. The CDI mouse model was induced as previously described (Hutton et al., 2014; Yan et al., 2014) with some modifications. The detailed procedure is shown in Figure 1A. Water containing antibiotics, comprising of kanamycin (0.8 mg/mL; Sigma), gentamicin (0.07 mg/mL; Sigma), colistin (0.1135 mg/mL; Sigma), MTZ (0.43 mg/mL; Sigma) and vancomycin (0.09 mg/mL; Sigma), was provided for 7 consecutive days starting on Day -9, and then replaced with autoclaved water from Day -2. A dose of clindamycin (10 mg/kg; Sigma) was given intraperitoneally at Day -1. The prepared toxigenic spores were administered to the model mice at a dose of 3 × 10^8^ colony forming units (cfu) by oral gavage from Day 0 for 3 consecutive days. The animals were observed every 4 h, mainly for the presence of diarrhea and death after the spore challenge. Body weight was measured daily from Day -9 to Day 2 (data after Day 3 not provided due to death of the mice). Cecal contents and cecum and colon tissue samples were collected, and *C. difficile* toxin was detected in supernatants of cecal contents, soon after the death of mice. The surviving mice were euthanized by CO_2_ inhalation at Day 7. Tissue samples including cecum and colon tissues were collected for histopathologic and immunohistochemical analysis and cecal contents were collected for 16S rRNA analysis. Mean optical density of the immunohistochemical images were qualified using Image J 1.51 software.

### Grouping

Mice were randomly assigned to seven groups (*n* = 6–8 mice/group), including Blank, CDI (*C. difficile* infection) model, BFT (*B. fragilis* prophylactic treatment 10^7^ cfu), BFT (10^8^ cfu), MTZ (metronidazole), BFC (*B. fragilis* control) and Saline groups. Mice in the Blank group were reared from beginning to end without any treatment. Mice in the CDI model group were treated as described in Section “Animals and CDI Model.” The BFT groups were used to explore the prophylactic effects of *B. fragilis* on preventing CDAD, and 1 × 10^7^ or 1 × 10^8^ cfu/day *B. fragilis* were given, respectively, to CDI model mice from D -9 to D2. Mice in the MTZ group were given MTZ (1 mg/day) orally from D0 to D5, as the positive control. Mice in the Saline group drank antibiotic water from D -9 to D -2 and were injected with clindamycin at D -1, to observe the effect of antibiotics alone on mouse gut microbiota. Mice in the BFC control group drank antibiotic water from D -9 to D -2 and were injected with clindamycin at D -1 and received 1 × 10^8^ cfu/day *B. fragilis* from the onset (D -9) to D2, to observe the effect of *B. fragilis* alone on mice with disturbed gut microbiota.

### Microbial DNA Extraction From Cecal Content and 16S rRNA Gene Sequencing

Microbial DNA was extracted from the mouse cecal content samples using a QIAamp Stool DNA Mini Kit (Qiagen, Hilden, Germany) following the manufacturer’s instructions. The V4 region of the 16S rRNA gene was amplified from the cecal DNA samples using the primer pair 515F/806R. All libraries were sequenced using the Ion S5^TM^ XL platform (Thermo Fisher, Waltham, MA, United States) by Nuohezhiyuan Co. (China).

Sequencing analysis was performed in the Quantitative Insights into Microbial Ecology framework (QIIME, Version 1.9.1) as described previously (Kuczynski et al., 2012). Briefly, low-quality bases were initially screened using Cutadapt (Version 1.9.1), and then pair reads were filtered using the UCHIME Algorithm. After filtering, we obtained a total of 2,412,968 raw reads from 28 cecal content samples. The average number of raw reads per sample was 86177 (±6690 SD), and the average number of clean reads per sample was 81207 (±6025 SD). Clean reads were clustered as OTUs based on the threshold of 97% identity, and representative sequences were annotated (top 10 were listed). The Chao diversity index and the number of observed species per sample were used as α-diversity metrics. β-diversity was calculated using unweighted UniFrac distances and represented in Non-Metric Multi-Dimensional Scaling (NMDS) analyses. The *T*-test method was used to analyze differences in species richness between groups.

Raw data for all samples used in this study have been deposited in the BioProject database at NCBI available as BioProject ID PRJNA505127, at http://www.ncbi.nlm.nih.gov/bioproject/505127.

### Histological Analysis

Cecum and colon tissue samples were fixed in 4% paraformaldehyde, embedded in paraffin, and cut into 4 μm sections. The sections were stained with hematoxylin-eosin and observed under an optical microscope (TE2000-U; Nikon, Tokyo, Japan). For immunohistochemical staining, the paraffin-embedded sections were deparaffinized, rehydrated, subjected to heat-induced antigen retrieval in citrate buffer, and blocked with PBS containing 1% (w/v) BSA. The sections were then incubated with anti-MUC-2, anti-ZO-1, anti-Occludin primary antibodies (Wuhan Service Biotechnology Co., Ltd., China) at a dilution of 1:100, followed by a biotinylated secondary antibody (Wuhan Service Biotechnology Co., Ltd.). Bound antibodies were visualized using 3,3′-diaminobenzidine as a substrate.

### Western Blotting

Mouse colon tissues were lysed using radio immunoprecipitation assay lysis buffer (Beyotime, Haimen, China), and tissue lysates were centrifuged at 14,000 ×*g* for 30 min. Supernatants were collected and mixed with 5× sodium dodecyl sulfate (SDS) sample buffer. The samples were separated by SDS-polyacrylamide gel electrophoresis using 12% acrylamide gels, and then transferred to polyvinylidene fluoride membranes (Millipore, Billerica, MA, United States). Following incubation with primary and secondary antibodies, protein bands were detected with Clarity^TM^ Western ECL Substrate (Bio-Rad, United States) and analyzed using the Biolmaging System (UVP, CA, United States). The following antibodies were used: rabbit anti-caspase 3, (Cat.: AF6311, Affinity, United States), rabbit anti-bcl-2(Cat.: AF6139, Affinity, United States), rabbit anti-bax (Cat.: AF0120, Affinity, United States), mouse anti-β-actin (Cat.: AC001-M, Dingguo, China).

### Adhesion Assays

HT-29 cells were seeded in 24-well plates at 1 × 10^5^ cells/well and incubated overnight. *B. fragilis* ZY-312 cells were harvested and adjusted to 5 × 10^8^ cfu/mL, and *C. difficile* cells were adjusted to 5 × 10^9^ cfu/mL. Bacterial cells in a volume of 20 μL were added to relevant wells in the following groups: A: positive control group, cells infected with *C. difficile* only; B: competition group, cells co-infected with *B. fragilis* and *C. difficile*; C: exclusion group, *B. fragilis* cells added for an hour prior to co-culture with *C. difficile*; D: substitution group, *C. difficile* cells added for an hour and prior to co-culture with *B. fragilis*. All cells were incubated anaerobically at 37°C for 2 h in total. After the incubation, non-adherent bacteria were removed by three washes with PBS and lysed with 1 mL of distilled water. The adherent *C. difficile* cells were serially diluted and spread on TSB plates containing 1 μg/mL clindamycin. The relative adhesion rates were calculated as follows: relative adhesion rate = adhesion rate/positive control adhesion rate × 100%.

### Transepithelial Electrical Resistance (TEER) Measurement

To determine the protective effect of *B. fragilis* ZY-312 on the permeability of colon cell monolayers, 2 × 10^4^ Caco-2 cells were seeded on 24-well transwell filters and cultured in medium containing 20% FBS for 21 days to allow polarization. The culture medium was replaced every other day. At Day 21, the cells had grown to 3 × 10^4^ cells in all wells and were washed three times with PBS. Next, 400 μL and 1 mL of culture medium was added into the upper and lower compartments, respectively. Six groups were created as follows: control group, cells cultured without any treatment; ZY-312 group, cells incubated with 3 × 10^7^ cfu *B. fragilis* ZY-312; *C. difficile* group, cells incubated with 3 × 10^6^ cfu *C. difficile* VPI 10463; competition group, exclusion group, and substitution group, as per the adhesion assays (see section “Western Blotting”). The TEER was measured with an EVOM2 (World Precision Instruments, Sarasota, FL, United States) in each transwell and TEER determined by the following equation: TEER = (target value - initial value) × 0.33.

### Immunofluorescence Assays for Zona Occludens-1 (ZO-1) and Muc-2

The expression level of the tight junction protein ZO-1 in Caco-2 cells and the mucin Muc-2 in HT-29 cells was determined by immunofluorescence. The groups were the same as those for the TEER assays (see section “Adhesion Assays”). Before observation, 4 × 10^4^ Caco-2 or HT-29 cells were seeded in 10-mm confocal dishes, allowed to adhere, and washed with PBS, before addition of bacterial cells as described above. The colon cells were infected with 4 × 10^7^ cfu *B. fragilis* and 4 × 10^6^ cfu *C. difficile* in each group. After the incubation, the cells were washed twice with PBS containing 0.1% Tween-20 (PBST), fixed with 200 μL of 2% paraformaldehyde for 30 min, washed three times with cold PBST, and blocked for 2 h. The blocking solution was composed of PBST containing 10% goat serum, 1% BSA and 3% glycine. Next, the cells were incubated with anti-ZO-1 or anti-Muc-2 antibodies (Abcam, Cambridge, United Kingdom) in PBST containing 1% BSA at 4°C overnight, washed three times with PBST, incubated with a secondary antibody conjugated with DyLight 488 (Abbkine, Wuhan, China) at room temperature for 1 h, washed three times with PBST, and incubated with 10 μg/ml DAPI for 10 min. After removal of the DAPI solution, one drop of fluorescence quenching agent was added to the cell surface and the cells were examined under a fluorescence microscope (TE2000-U; Nikon).

### Periodic Acid-Schiff (PAS) Staining for Muc-2 Protein

Periodic acid-Schiff staining reagent (SenBeiJia, Nanjing, China) was used to visualize Muc-2 in HT-29 cell monolayers according to the manufacturer’s protocol. Briefly, 1 × 10^5^ cells were seeded in 24-well plates and infected with 1 × 10^7^ cfu *C. difficile* and 1 × 10^8^ cfu *B. fragilis* for 2 h. The groups and treatments were as described in Section “Western Blotting.” After the treatment, the cells were washed three times with PBS, incubated with 300 μL of PAS solution for 15 min at room temperature, washed, oxidized with 200 μL/well periodic acid solution for 20 min, washed, incubated with 200 μL/well Schiff reagent for 30 min, and washed twice with sodium sulfite solution. The cells were counterstained with hematoxylin and observed under an optical microscope (IMT-2, Olympus, Tokyo, Japan).

### Cell Apoptosis Assay

A flow cytometry method was used to examine the effects of *B. fragilis* ZY-312 on *C. difficile*-induced apoptosis of HT-29 cells. Briefly, 5 × 10^5^ cells were inoculated into 35-mm culture dishes and treated for 2 h in different groups as described in Section “Western Blotting” (multiplicity of infection: 1000 for *B. fragilis* and 100 for *C. difficile*). All cells were harvested by centrifugation, stained with Annexin V-FITC and PI, and analyzed by flow cytometry (FACSCalibur; BD Biosciences). Staining was also performed on Vero and IEC-6 cells to observe the protective effects of *B. fragilis* ZY-312. These cells were treated as described above, stained with Annexin V-FITC and PI, and observed under a fluorescence microscope. Unstained cells were observed under an optical microscope.

### Statistical Analysis

Data are presented as the mean ± SEM, unless otherwise indicated. All cell experiments were performed three times independently and each experiment was performed in triplicate. Statistical analyses for the significance of differences in data were performed by a two-tailed *t*-test, repeated-measures analysis of variance, or one-way analysis of variance where appropriate. Values of *p* < 0.05 were considered statistically significant.

## Results

### *C. difficile* VPI 10463 Spores Successfully Induced CDAD, Gut Microbiota Disorder, Gut Barrier Disruption, and Death

**FIGURE 2:**
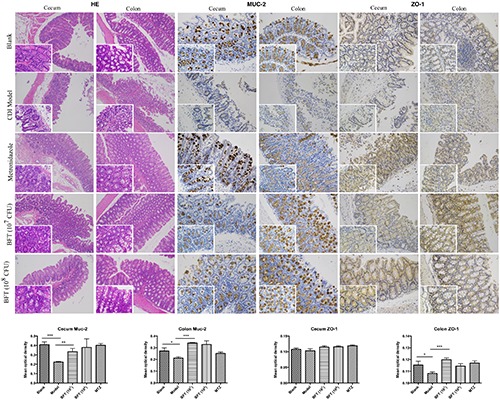
*B. fragilis* ZY-312 improves gut barrier integrity and function in the CDI mouse model. Representative images of hematoxylin-eosin stained cecum and colon tissue samples from all groups are shown. Images showing representative immunohistochemical staining of Muc-2 and ZO-1 protein located in cecal and colon tissues in the Blank, CDI model, MTZ, and BFT groups are also shown. Mice in the Blank group were reared parallelly without any treatment. Mice in the CDI model group drank water with antibiotics including kanamycin (0.8 mg/mL), gentamicin (0.07 mg/mL), colistin (0.1135 mg/mL), metronidazole (0.43 mg/mL), and vancomycin (0.09 mg/mL) from D –9 to D –2, intraperitoneally injected with clindamycin (10 mg/kg) at D –1, and orally challenged with 3 × 10^8^ cfu of *C. difficile* spores from D0 to D2. Mice in the MTZ group were treated with metronidazole (1 mg/day) from the day of *C. difficile* spore challenge (D0) to D5. Mice in the BFT groups were CDI model mice prophylactically treated with 1 × 10^7^ or 1 × 10^8^ cfu/day *B. fragilis* from the onset (D –9) to the end of CDI modeling (D2). Data of mean optical density are shown as means ± SEM. ^∗^*p* < 0.05, ^∗∗^*p* < 0.01, ^∗∗∗^*p* < 0.001, by unpaired *t*-test.

**FIGURE 3:**
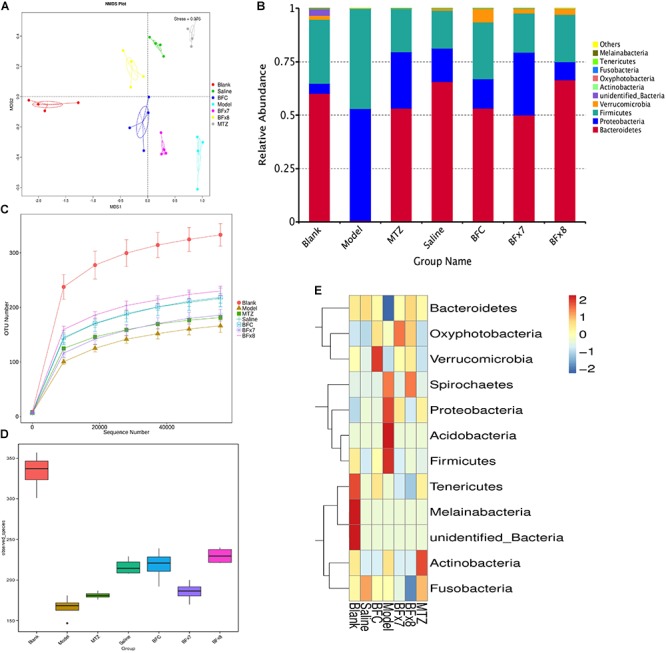
*Bacteroides fragilis* ZY-312 regulates gut microbiota in the CDI mouse model. **(A)** Non-Metric Multi-Dimensional Scaling analyses based on OTUs in the Blank, Model, MTZ, Saline, BFC, BF × 7, and BF × 8 groups. **(B)** Relative abundance of the top 10 phyla in the gut microbiota of all groups. **(C)** Rarefaction curve showing the relative abundance of OTUs in all groups. **(D)** Observed species in all groups. **(E)** Cluster heat map indicating the richness of the top 12 phyla in all groups (Stress values < 0.2 indicates significant differences between samples). Mice in the Blank group were reared parallelly without any treatment. Mice in the CDI model group drank water with antibiotics including kanamycin (0.8 mg/mL), gentamicin (0.07 mg/mL), colistin (0.1135 mg/mL), metronidazole (0.43 mg/mL), and vancomycin (0.09 mg/mL) from D –9 to D –2, were intraperitoneally injected clindamycin (10 mg/kg) at D –1, orally challenged with 3 × 10^8^ cfu of *C. difficile* spores from D0 to D2. Mice in the MTZ group were treated with metronidazole (1 mg/day) from the day of spore challenge (D0) to D5. Mice in the BFT groups were CDI model mice prophylactically treated with 1 × 10^7^ cfu/day (BF × 7 group) or 1 × 10^8^ cfu/day (BF × 8 group) *B. fragilis* from the onset (D –9) to the end of CDI modeling (D2). Mice in the Saline group drank antibiotic water from D –9 to D –2 and were injected with clindamycin at D –1, to observe the effect of antibiotics alone on mice gut microbiota. Mice in the BFC control group drank antibiotic water from D –9 to D –2, were injected with clindamycin at D –1 and received 1 × 10^8^ cfu/day *B. fragilis* from the onset (D –9) to D2, to observe the effect of *B. fragilis* alone on mice with antibiotic-induced dysbiosis.

In this study we successfully induced a CDI model by challenging antibiotic-treated mice with *C. difficile* spores. All CDI model mice (100%) developed serious diarrheic symptoms (Table 1) and weight loss (Supplementary Figure S1C) soon after spore challenge, and 87.5% of the mice had died at Day 2 (Figure 1B). *C. difficile* toxin was detected in the cecal contents of all model mice (Supplementary Figure S1B). Significant histopathological damage was observed in the tissue samples (Figure 1C), especially in the gut epithelium (Figure 2). The colon structure was destroyed and enterocytes had died, with obvious inflammatory cell infiltration, digestive gland destruction, and goblet cell loss, compared with the vehicle control group. Muc-2 and ZO-1 protein loss was confirmed by immunohistochemical analysis. In 16S rRNA analyses, the gut microbiota of antibiotic-treated mice was significantly perturbed after spore challenge (Supplementary Figures S4, S5). The OTU number (Figure 3C) was dramatically decreased and the relative abundance (Figure 3B) remained only two phyla, *Proteobacteria* and *Firmicutes*, while the richness of *Bacteroidetes* decreased to zero, consistent with a previous report (Wang et al., 2017). The cluster heat map further revealed that most of the altered phyla (Figure 3E) and species (Supplementary Figure S4) in the cecal contents belonged to the *Firmicutes* phylum.

**Table 1:** Incidence of *Clostridium difficile*-induced diarrhea and effect of treatment.

Group	D1	D2	D3
Blank	0	0	0
CDI model	87.5% (7/8)	100% (8/8)	0
Metronidazole	0	66.7% (4/6)	20% (1/5)
Saline control	0	0	33.3% (2/6)
BFC	0	50% (3/6)	0
ZY-312 × 10^7^	0	37.5% (3/8)	0
ZY-312 × 10^8^	75% (6/8)	37.5% (3/8)	12.5% (1/8)


*Clostridium difficile spores were used to challenge the antibiotic treated mice at D0, and D1, D2. D1, D2 and D3 means the first, second, and third day after D0, respectively.*

### *B. fragilis* ZY-312 Reduces Diarrhea and Death, Likely by Regulating Gut Microbiota and Protecting the Gut Barrier

In this study, we verified the protective effects of *B. fragilis* in a CDI mouse model. First, we tested the safety of a high dose (1 × 10^8^ cfu) of *B. fragilis* in antibiotic-treated mice, no mice died in the BFC group and 50% displayed mild diarrhea at Day 2, but soon recovered by Day 3 (Table 1 and Figure 2). Histopathological and immunohistochemical analyses provided consistent results (Supplementary Figure S2). Next, we prophylactically treated the CDI mice with *B. fragilis* (1 × 10^7^ and 1 × 10^8^ cfu/day) and impressive protective effects were observed. All mice prophylactically treated with *B. fragilis* displayed much lower morbidity (Table 1) and mortality (Figure 1B) compared with the CDI model mice. The expressions of Muc-2 and ZO-1 proteins (Figure 2) were better preserved, and expressions of the apoptosis proteins (Supplementary Figure S2D) were inhibited in intestinal cells, indicating that *B. fragilis* can protect the gut epithelium of CDI model mice. Furthermore, 16S rRNA analyses revealed that *B. fragilis* was sufficient to regulate the cecal microbiota in the CDI model mice by increasing the relative abundance and OTU number, and rebalancing the composition (Figure 3 and Supplementary Figures S4, S5). A NMDS analysis (Figure 3A) demonstrated that our samples were qualified and acceptable, because the distributions of the inter-group samples were scattered much more discretely than those of the intra-group samples. Notably, the distribution of the microbiome in the MTZ group was obviously different from that in the BFT groups, indicating that the mechanism of MTZ treatment was different from *B. fragilis*. Similarly, the relative abundance of *Verrucomicrobia* (Figure 3B) and of its representative species *Akkermansia muciniphila* (Supplementary Figure S5B) in the cecum of antibiotic-treated mice (with or without spore challenge) was significantly increased after *B. fragilis* prophylactic treatment of CDI model mice.

### *B. fragilis* ZY-312 Inhibits *C. difficile* Adherence to HT-29 Cells

**FIGURE 4:**
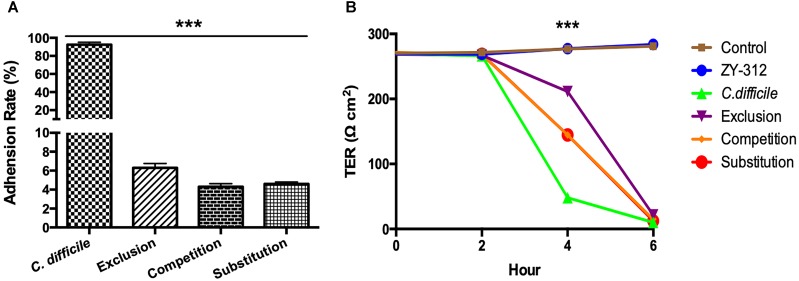
*Bacteroides fragilis* ZY-312 inhibits *C. difficile* adherence and improves TEER. **(A)** Adherence to HT-29 cells in *C. difficile*, Exclusion, Competition and Substitution groups. *C. difficile* group, 1 × 10^5^ HT-29 cells were infected with 1 × 10^7^ cfu *C. difficile*; Competition group, 1 × 10^5^ HT-29 cell were co-infected with 1 × 10^8^ cfu *B. fragilis* and 1 × 10^7^ cfu *C. difficile*; Exclusion group, cells were infected with *B. fragilis* for the first hour and *C. difficile* added for the second hour; Substitution group, *C. difficile* were added for the first hour and *B. fragilis* added for the second hour. The cells were incubated at 37°C under anaerobic conditions for 2 h in total. **(B)** Transepithelial electrical resistance (TEER) values for Caco-2 cells in Control, *B. fragilis* ZY-312, *C. difficile*, Exclusion, Competition and Substitution groups. Control group, 2 × 10^4^ Caco-2 cells were cultured without treatment. ZY-312 group, cells were incubated with 3 × 10^7^ CFU *B. fragilis* ZY-312. *C. difficile* group, cells were incubated with 3 × 10^6^ CFU *C. difficile*. Competition group, cells were co-infected with 3 × 10^7^ CFU *B. fragilis* and 3 × 10^6^ CFU *C. difficile*. Exclusion group, *B. fragilis* infected cells for the first hour and *C. difficile* for the second hour; Substitution group, *C. difficile* infected cells for the first hour and *B. fragilis* for the second hour. Data are shown as means ± SEM. ^∗∗∗^*p* < 0.001 by ANOVA.

In the present study, we examined whether *B. fragilis* could influence the adherence of *C. difficile* to colon cells, because colonization is a vital step for initiation of CDI pathogenesis *in vivo*. We found that *B. fragilis* significantly reduced the adherence of *C. difficile* to HT-29 cells (Figure 4A). All cells in the groups incubated with *B. fragilis* exhibited obvious resistance to *C. difficile* adherence, demonstrating that *B. fragilis* is capable of inhibiting *C. difficile* adherence to colon cells *in vitro*.

### *B. fragilis* ZY-312 Attenuates *C. difficile*-Induced Reduction in TEER in Caco-2 Cells

As shown in Figure 4B, *C. difficile* induced a dramatic reduction in TEER of colon cell monolayers soon after infection, indicating that it is sufficiently toxic to cause gut barrier damage and increase gut permeability *in vitro*. A significant difference in the TEER value was observed at 4 h between the *C. difficile*-treated groups and the other groups (*p* < 0.001). Among the groups, the exclusion group presented the strongest protection, suggesting that colon cells pretreated with *B. fragilis* exhibit the best improvement of gut barrier permeability.

### *B. fragilis* ZY-312 Attenuates the Loss of Tight Junction Proteins and Mucins in Colon Cells Induced by *C. difficile*

**FIGURE 5:**
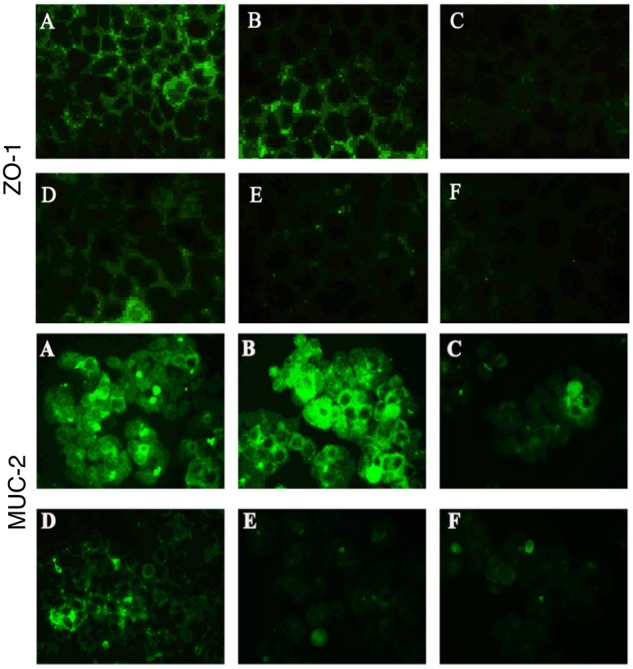
*Bacteroides fragilis* ZY-312 increases expression of the tight junction protein ZO-1 and mucin MUC-2 in colon cells infected with *C. difficile*. Representative images for immunofluorescence staining of tight junction protein ZO-1 (top) in Caco-2 cells and mucin Muc-2 (middle) in HT-29 cells are shown for all groups. **(A)** Blank control group, 4 × 10^4^ Caco-2 or HT-29 cells were cultured without treatment. **(B)** *B. fragilis* group, cells were incubated with 4 × 10^7^ cfu *B. fragilis*. **(C)** *C. difficile* group, cells were incubated with 4 × 10^6^ cfu *C. difficile*. **(D)** Exclusion group, cells were infected with 4 × 10^7^ cfu *B. fragilis* for the first hour and 4 × 10^6^ cfu *C. difficile* for the second hour. **(E)** Competition group, cells were co-infected with *B. fragilis* and *C. difficile*. **(F)** Substitution group, *C. difficile* was added for the first hour and *B. fragilis* added at the second hour. The cells were incubated at 37°C under anaerobic conditions for 2 h.

In the present study, we performed immunofluorescence staining to visualize ZO-1 and Muc-2 proteins and determine the protective effects of *B. fragilis* on tight junctions and barrier function in the epithelial system (Figure 5). The data showed strong green fluorescence in the control group and *B. fragilis*-treated group, indicating that ZO-1 protein was present in the intercellular spaces. In contrast, fluorescence of ZO-1 protein was almost absent in cells treated with *C. difficile*, demonstrating that the *C. difficile*-induced damage to the gut barrier probably involves tight junction disruption. Although *B. fragilis* did not provide obvious protection against loss of ZO-1 protein in the competition group and substitution group, it did offer partial protection in the exclusion group.

Similarly, *C. difficile* significantly compromised MUC-2 expression and the reduction was attenuated by pretreatment with *B. fragilis* (Figure 5). The consistent results for PAS staining (Supplementary Figure S3) in HT-29 cells further confirmed the protective effects of *B. fragilis* on MUC-2 expression in the *in vitro* CDI model.

### *B. fragilis* ZY-312 Inhibits Colon Cell Apoptosis Induced by *C. difficile*

**FIGURE 6:**
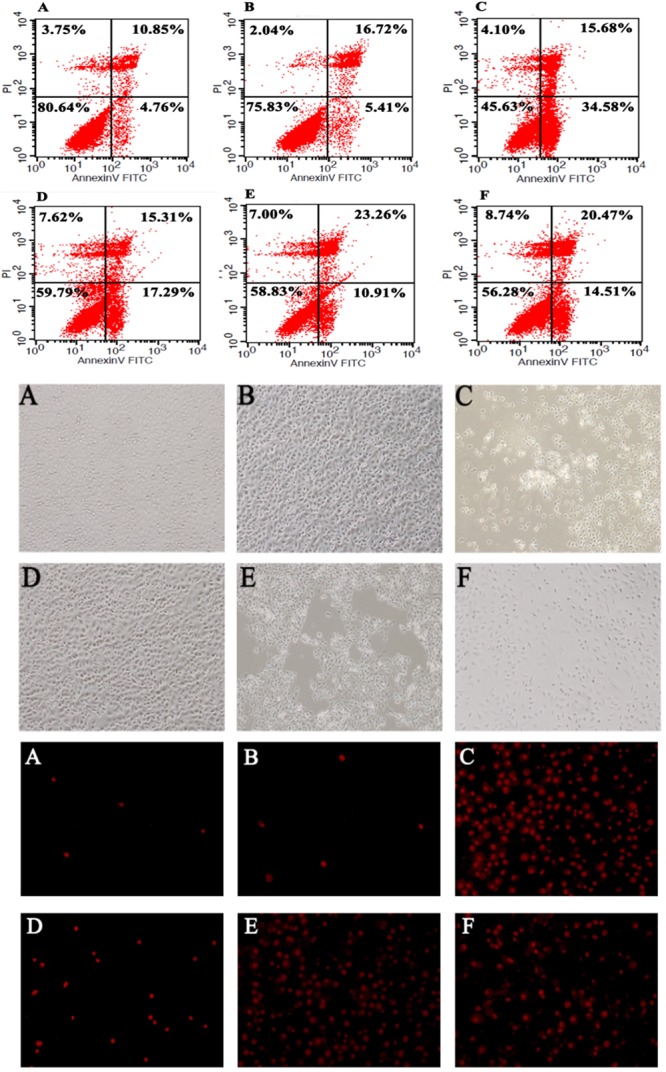
*Bacteroides fragilis* ZY-312 inhibits colon cell apoptosis induced by *C. difficile*. Flow cytometry analyses **(top)** of HT-29 cells in all groups are shown. The lower right quadrant represents colon cells in early apoptosis. Microscopic observations **(middle)** of IEC-6 cell morphology and viability and PI staining **(bottom)** of IEC-6 cells in all groups are shown. **(A)** Blank control group, 5 × 10^5^ HT-29 or IEC-6 cells were cultured without treatment. **(B)** *B. fragilis* group, cells were incubated with 5 × 10^8^ cfu *B. fragilis*. **(C)** *C. difficile* group, cells were incubated with 5 × 10^7^ cfu *C. difficile*. **(D)** Exclusion group, cells were infected with 5 × 10^8^ cfu *B. fragilis* for the first hour and 5 × 10^7^ cfu *C. difficile* for the second hour. **(E)** Competition group, cells were co-infected with *B. fragilis* and *C. difficile*. **(F)** Substitution group, cells were infected with *C. difficile* for the first hour and *B. fragilis* for the second hour. The cells were incubated at 37°C under anaerobic conditions for 2 h.

According to the flow cytometry analysis (Figure 6), *C. difficile* induced significant early apoptosis of HT-29 cells (lower right quadrant), which was partly inhibited by *B. fragilis*. Specifically, pretreatment, co-incubation, and post-treatment with *B. fragilis* were all able to reduce the early apoptosis of HT-29 cells, demonstrating that *B. fragilis* improved cell survival in the *in vitro* CDI model. Microscopic observation of the IEC (Figure 6) and Vero (Supplementary Figure S3) cell morphology and viability provided consistent results. Treatment with *B. fragilis* significantly attenuated the damage, especially when the cells were pretreated.

## Discussion

Gut microbiota have received extensive attention in recent years, as their relationship with human health is far closer than previously expected. *Clostridium difficile* (*C. difficile*) is currently the single leading cause of inpatient infections and has become a public health threat due to its high morbidity and mortality rates (Kelly and LaMont, 2008). Antibiotic overuse, which disrupts the normal healthy gut microbiota of the host, is a huge risk factor for *C. difficile* infection (CDI) in the clinical setting. FMT is regarded as a potential bacteriotherapy for refractory CDI patients, especially for patients with recurrent disease (van Nood et al., 2013). A study reported that 13 of 16 patients recovered from CDI after performing duodenal infusion of donor feces just once. The bacterial diversity of the recovered patients increased and their microbiota composition became similar to the donors’, with an increase in the relative abundance in *Bacteroidetes* species and a relative decrease in the abundance in *Proteobacteria* species (van Nood et al., 2013). However, there are several factors regarding FMT remaining to be settled, including the standards for a healthy donor, the storage of feces, and the risk of infusing opportunistic pathogens (Kelly and Tebas, 2018). To address these issues, Lawley et al tried to create a simpler and more targeted bacteriotherapy for CDI. They produced a simple mixture of six phylogenetically diverse intestinal bacteria which was able to clear the supershedding state of *C. difficile* 027/BI infected mice (Lawley et al., 2012), through helping the host re-establish a health-associated microbiota. A novel species of *Bacteroides* was included in that mixture, although it did not work in isolation nor mixed with *Lactobacillus*.

In this study, a single strain of *B. fragilis* was not able to rebuild a total health-associated microbiota net in CDI mice (Figure 3 and Supplementary Figure S4), although we did observe a significant increase in survival rate of the CDI mice prophylactically treated with *B. fragilis*. As a member of the commensal microbiota, *B. fragilis* plays an important role in microbe-host homeostasis, including regulation of immune system maturation and maintenance of immune tolerance (Round and Mazmanian, 2010). Moreover, *B. fragilis* protects mice from experimental colitis (Mazmanian et al., 2008) via a commensal symbiosis factor expressed in the outer membrane vesicles (Shen et al., 2012). This symbiosis factor was PSA, a zwitterionic polysaccharide, which protects the inflamed epithelium through boosting interleukin (IL)-10-producing CD4^+^ T cells and suppressing proinflammatory IL-17-producing CD4^+^ T cells (Mazmanian et al., 2008). Expansion and activation of Treg cells is important for maintain homeostasis after epithelial damage (Geuking et al., 2011), but PSA is not the only symbiosis factor produced by *B. fragilis* which influences epithelial homeostasis. Another study (Lee et al., 2013) revealed that *B. fragilis* has evolved to encode a special but highly conserved polysaccharide utilization locus for stable gut colonization. This genetic locus was named the commensal colonization factor (CCF), which not only helps *B. fragilis* penetrate the mucus and reside deep in colon crypt, but also creates a reservoir for resilience after enteric infections or antibiotic exposure. Previously we tracked the inhibition from *B. fragilis* to *Vibrio parahaemolyticus* infection in mice (Li et al., 2017), indicating *B. fragilis* also performed as the first-line defender to resist enteric pathogens. Here we observed obvious inhibition of *C. difficile* adherence by *B. fragilis* *in vitro* (Figure 4), and speculated that this might be an important preventive mechanism *in vivo* as well.

Impressively, another report (Hsiao et al., 2013) demonstrated that *B. fragilis* was able to ameliorate autism-related gastrointestinal and behavioral abnormalities in an autism mouse model, through restoration of gut barrier integrity and correction of abnormal microbiome composition. Our recent report (Zhang et al., 2018) further discovered that *B. fragilis* significantly ameliorated AAD-related diarrhea symptoms by increasing the abundance of specific commensal microbiota and restoring intestinal barrier function. As we know, *C. difficile* toxins, including Toxin A and Toxin B, mainly target intestinal epithelium through disrupting the cell cytoskeleton and inducing necrosis (Pothoulakis, 2000), thereby damaging colonic epithelial integrity. Therefore, we speculated that *B. fragilis* prevention of CDAD probably involved the improvement of gut barrier integrity. The results of Muc-2 and ZO-1 expression in *in vivo* (Figure 2) and *in vitro* (Figure 5) experiments suggested that *B. fragilis* was able to restore expression of mucins and tight junction proteins in epithelial cells during CDI.

Microbiome regulation was considered another possible mechanism by which *B. fragilis* prevents CDI. *B. fragilis* obviously increased intestinal bacterial diversity and commensal bacteria relative abundance in CDI mice (Figure 3 and Supplementary Figures S4, S5). A specific phenomenon was observed whereby the relative abundance of the *Verrucomicrobia* phylum was increased after *B. fragilis* prophylactic treatment group (Supplementary Figure S5). Comprising 98% of the *Verrucomicrobia* phylum, *A. muciniphila* is a Gram-negative, mucin-utilizing commensal bacterium that resides in the mucus layer of the human intestinal tract. It has been reported that *A. muciniphila* has evolved to specialize in degrading and utilizing mucus to provide a source of carbon and nitrogen. Belzer et al. (2017) co-cultured *A. muciniphila* with the non-mucus-degrading butyrate-producing bacteria *Eubacterium hallii* and found syntrophic growth and butyrate production. This study reminds us of the probability that there might also exist bacterial cross-feeding interactions between *A. muciniphila* and *B. fragilis*, since *B. fragilis* is the major source of short-chain fatty acids in the intestinal tract (Wang et al., 2017). Briefly, cross-feeding is one microorganism utilizing the end products or carbohydrate breakdown products offered by another microorganism (Rios-Covian et al., 2016), which is common among gut microbiota. The exact mechanisms of the interactions between *B. fragilis*, *A. muciniphila*, and the intestinal epithelium remain to be clearly established, but the maintenance and growth of *A. muciniphila* after *B. fragilis* pretreatment is believed to take part in restoring gut barrier integrity during *C. difficile* pathogenesis.

With the initial identification of an inverse relationship between *B. fragilis* and *C. difficile* (Goldberg et al., 2014), and based on the theory of gut barrier disruption by CDI and protection by *B. fragilis*, we have proven that *B. fragilis* prophylactic treatment can ameliorate morbidity and mortality in a CDI mouse model. This is likely through inhibiting *C. difficile* colonization, modulating gut microbiota and alleviating barrier destruction, thus relieving epithelial stress and pathogenic colitis. The present findings demonstrated the direct protection against CDI provided by a single strain of commensal bacteria, which lay the foundations for exploring the optimal bacteriotherapy for CDI prophylaxis and treatment. They also provide deep insight into microbe-microbe and microbe-epithelium interactions. However, further studies are needed to clarify the molecular mechanisms involved in the protective roles of *B. fragilis* in gut barrier maintenance and microbe-microbe interactions.

## Author Contributions

HD performed the experiments with bacteria and mice, analyzed the data, and wrote the manuscript. SY performed the experiments with bacteria and mice and analyzed the data. KQ and ZZ performed the experiments with mice. YZ performed the experiments with bacteria and cells, analyzed the data, and contributed to revising the manuscript. YL and YW provided the bacteria and contributed to revising the manuscript. YB contributed to revising the manuscript. HF and XZ designed the experiments, analyzed the data, and contributed to revising the manuscript. FZ provided overall directions and contributed to revising the manuscript.

## Conflict of Interest Statement

*B. fragilis* ZY-312 belongs to Guangzhou ZhiYi Biotechnology Co., Ltd. Any use of *B. fragilis* ZY-312 without the permission of Guangzhou ZhiYi Biotechnology Co., Ltd. is illegal. YL and YW were employed by Guangzhou ZhiYi Biotechnology Co., Ltd. The remaining authors declare that the research was conducted in the absence of any commercial or financial relationships that could be construed as a potential conflict of interest.

## Abbreviations

AAD: antibiotic-associated diarrhea
BFT: Bacteroides fragilis treatment
BHI: brain heart infusion
BSA: bovine serum albumin
CDAD: Clostridium difficile associated disease
CDI: Clostridium difficile infection
DAPI: 2-(4-Amidinophenyl)-6-indolecarbamidine
DNA: deoxyribonucleic acid
FBS: fetal bovine serum
FITC: fluoresceine isothiocyanate
FMT: fecal microbiota transplantation
MTZ: metronidazole
NMDS: non-metric multidimensional scaling
OTU: operational taxonomic unit
PAS: Periodic acid-Schiff
PBS: phosphate-buffered saline
PBST: phosphate-buffered saline containing 0.1% Tween-20
PCR: polymerase chain reaction
PI: propidium iodide
RPMI: roswell park memorial institute
rRNA: ribosomal ribonucleic acid
SEM: standard error of mean
TEER: transepithelial electrical resistance
TSB: tryptone soy broth
ZO-1: zona occludens-1
