# Corrigendum: *Bacteroides fragilis* Prevents *Clostridium difficile* Infection in a Mouse Model by Restoring Gut Barrier and Microbiome Regulation

**DOI:** 10.3389/fmicb.2019.00601

**Published:** 2019-04-02

**Authors:** Huimin Deng, Siqi Yang, Yucheng Zhang, Kai Qian, Zhaohui Zhang, Yangyang Liu, Ye Wang, Yang Bai, Hongying Fan, Xinmei Zhao, Fachao Zhi

**Affiliations:** ^1^Guangdong Provincial Key Laboratory of Gastroenterology, Department of Gastroenterology, Institute of Gastroenterology of Guangdong Province, Nanfang Hospital, Southern Medical University, Guangzhou, China; ^2^Guangzhou ZhiYi Biotechnology Co., Ltd., Guangzhou, China; ^3^Guangdong Provincial Key Laboratory of Tropical Disease Research, School of Public Health, Southern Medical University, Guangzhou, China

**Keywords:** next-generation probiotic, gut barrier, gut microbiota, *Clostridium difficile*, commensal bacteria

In the original article, there was a mistake in the Supplementary Figure 3 as published. The same Figure 3 used in the original article was also used for Supplementary Figure 3. The corrected Supplementary Figure 3 appears below.

**Supplementary Figure 3:**
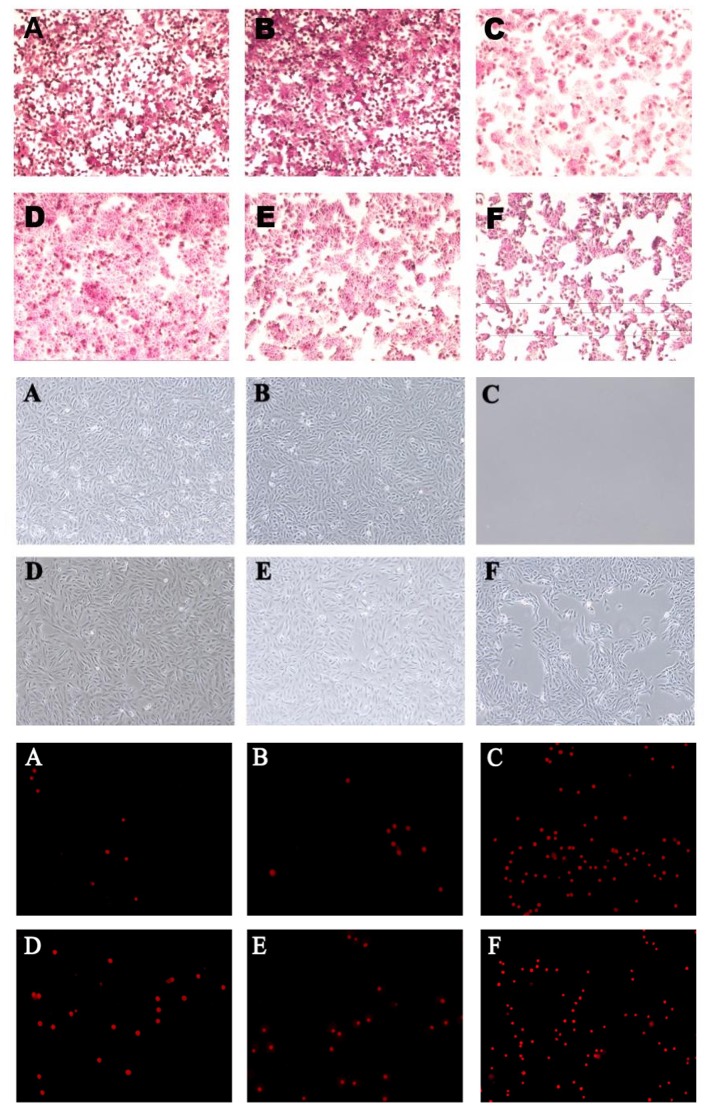
*B. fragilis* ZY-312 inhibits colon cell apoptosis induced by *C. difficile*. Representative images of PAS staining (top) for Muc-2 protein visualization in HT-29 cell monolayers are shown for all groups. Microscopic observations (middle) of Vero cell morphology and viability and PI staining (bottom) of Vero cells in all groups are shown. **(A)** Blank control group, 5 × 10^5^ HT-29 or Vero cells were cultured without treatment; **(B)** *B. fragilis* group, cells were incubated with 5 × 10^8^ cfu *B. fragilis*; **(C)** *C. difficile* group, cells were incubated with 5 × 10^7^ cfu *C. difficile*; **(D)** Exclusion group, cells were infected with 5 × 10^8^ cfu *B. fragilis* for the first hour and 5 × 10^7^ cfu *C. difficile* for the second hour; **(E)** Competition group, cells were co-infected with *B. fragilis* and *C. difficile*; **(F)** Substitution group, cells were infected with *C. difficile* for the first hour and *B. fragilis* for the second hour. The cells were incubated at 37°C under anaerobic conditions for 2 h in total.

The authors apologize for this error and state that this does not change the scientific conclusions of the article in any way. The original Supplementary Material has been updated.

