# Supplementary material for: Bacteroides fragilis Prevents Clostridium difficile Infection in a Mouse Model by Restoring Gut Barrier and Microbiome Regulation
Source: Front Microbiol. 2018 Dec 21;9:2976. doi: 10.3389/fmicb.2018.02976 (PMC6308121; doi:10.3389/fmicb.2018.02976)
Supplement: Supplementary file 1 [file Data_Sheet_1.pdf]

Fig. S1. ***C. difficile*** **infection model is established.**

(A) Observations of *C. difficile* VPI 10463 spores (1000× magnification) under an electron microscope after Gram-staining. **(B)** Confirmation of antigen and toxin in the supernatant of cecal contents of *C. difficile* model mice. **(C)** Body weight of mice in the Blank, CDI model, MTZ, Saline, BFC, and BFT groups. Mice in the Blank group were reared parallelly without any treatment; Mice in the CDI model group drank water with antibiotics from D -9 to D -2, were intraperitoneally injected with clindamycin at D -1, and challenged with 3×10^{8} cfu of *C. difficile* spores orally from D0 to D2. Mice in the MTZ group were treated with metronidazole (1 mg/day) from the day of spore challenge (D0) to D5. Mice in the BFT groups were CDI model mice prophylactically treated with 1×10^{7} or 1×10^{8} cfu/day *B. fragilis* from the onset (D -9) to the end of CDI modeling (D2). Mice in the Saline group drank antibiotic water from D -9 to D -2 and were injected with clindamycin at D -1, to observe the effect of antibiotics alone on mice gut microbiota. Mice in the BFC control group drank antibiotic water from D -9 to D -2, were injected with clindamycin at D -1 and received 1×10^{8} cfu/day *B. fragilis* from the onset (D -9) to D2, to observe the effect of *B. fragilis* on mice with dysbiosis.

Fig. S2. ***B. fragilis*** **ZY-312 improves gut barrier integrity and function in the CDI mouse model.**

(A) Images showing representative immunohistochemical staining of Occludin protein located in cecal and colon tissues in the Blank, CDI model, MTZ, BFT, BFC and Saline groups are also shown. Mice in the Blank group were reared parallelly without any treatment. Mice in the CDI model group drank water with antibiotics including kanamycin (0.8 mg/mL), gentamicin (0.07 mg/mL), colistin (0.1135 mg/mL), metronidazole (0.43 mg/mL) and vancomycin (0.09 mg/mL) from D -9 to D -2, intraperitoneally injected with clindamycin (10 mg/kg) at D -1, and orally challenged with 3×10^{8} cfu of *C. difficile* spores from D0 to D2. Mice in the MTZ

group were treated with metronidazole (1 mg/day) from the day of *C. difficile* spore challenge (D0) to D5. Mice in the BFT groups were CDI model mice prophylactically treated with 1×10^7 or 1×10^8 cfu/day *B. fragilis* from the onset (D -9) to the end of CDI modeling (D2). Mice in the Saline group drank antibiotic water from D -9 to D -2 and were injected with clindamycin at D -1, to observe the effect of antibiotics alone on mice gut microbiota. Mice in the BFC control group drank antibiotic water from D -9 to D -2, were injected with clindamycin at D -1, and received 1×10^8 cfu/day *B. fragilis* from the onset (D -9) to D2, to observe the effect of *B. fragilis* on mice with antibiotic-induced dysbiosis. **(B)** Mean optical density of cecum occludin proteins. **(C)** Mean optical density of colon occludin proteins. **(D)** Expressions of caspase-3, pro-caspase-3, bcl-2, bax in intestinal tissues of Blank, Model, MTZ, BFT (10^7cfu) and BFT (10^8cfu) group. **(E)** Representative images of hematoxylin-eosin stained cecum and colon tissue samples, representative immunohistochemical staining of Muc-2, ZO-1 protein located in cecal and colon tissues in the BFC and Saline groups are also shown. Data are shown as means ± SEM. * p <0.05 , by unpaired t test.

Fig. S3. *B. fragilis* ZY-312 inhibits colon cell apoptosis induced by *C. difficile*. Representative images of PAS staining (top) for Muc-2 protein visualization in HT-29 cell monolayers are shown for all groups. Microscopic observations (middle) of Vero cell morphology and viability and PI staining (bottom) of Vero cells in all groups are shown. **(A)** Blank control group, 5×10^5 HT-29 or Vero cells were cultured without treatment; **(B)** *B. fragilis* group, cells were incubated with 5×10^8 cfu *B. fragilis*; **(C)** *C. difficile* group, cells were incubated with 5×10^7 cfu *C. difficile*; **(D)** Exclusion group, cells were infected with 5×10^8 cfu *B. fragilis* for the first hour and 5×10^7 cfu *C. difficile* for the second hour; **(E)** Competition group, cells were co-infected with *B. fragilis* and *C. difficile*; **(F)** Substitution group, cells were infected with *C. difficile* for the first hour and *B. fragilis* for the second hour. The cells were incubated at 37°C

under anaerobic conditions for 2 h in total.

Fig. S4. *B. fragilis* ZY-312 regulates gut microbiota in the CDI mouse model.

Raw data of 16S rRNA sequencing analysis from the Blank, Model, MTZ, Saline, BFC, BF×7, BF×8 groups. **(A)** Observed species number. **(B)** Relative abundance of top 10 phlya. **(C)** Cluster heatmap of top 35 genus. Mice in the Blank group were reared parallelly without any treatment. Mice in the CDI model group drank water with antibiotics from D -9 to D -2, were intraperitoneally injected with clindamycin (10 mg/kg) at D -1, and orally challenged with 3×10^8 cfu of *C. difficile* spores from D0 to D2. Mice in the MTZ group were treated with metronidazole (1 mg/day) from the day of spore challenge (D0) to D5. Mice in the BFT groups were CDI model mice prophylactically treated with 1×10^7 cfu/day (BF×7 group) or 1×10^8 cfu/day (BF×8 group) *B. fragilis* from the onset (D -9) to the end of CDI modeling (D2). Mice in the Saline group drank antibiotic water from D -9 to D -2, and were injected with clindamycin at D -1 to observe the effect of antibiotics alone on mice gut microbiota. Mice in the BFC control group drank antibiotic water from D -9 to D -2, were injected with clindamycin at D -1, and received 1×10^8 cfu/day *B. fragilis* from the onset (D -9) to D2, to observe the effect of *B. fragilis* on mice with antibiotic-induced dysbiosis.

Fig. S5. *B. fragilis* ZY-312 increases the relative abundance of *A. muciniphila* in the CDI mouse model.

(A) Relative abundance of the top 10 families in the gut microbiota in all groups. **(B)** Relative abundance of *Akkermansia muciniphila* was found as a significant biomarker among groups.
