# Supplementary figures and images for: Bacteroides fragilis Prevents Clostridium difficile Infection in a Mouse Model by Restoring Gut Barrier and Microbiome Regulation
Source: Front Microbiol. 2018 Dec 21;9:2976. doi: 10.3389/fmicb.2018.02976 (PMC6308121; doi:10.3389/fmicb.2018.02976)

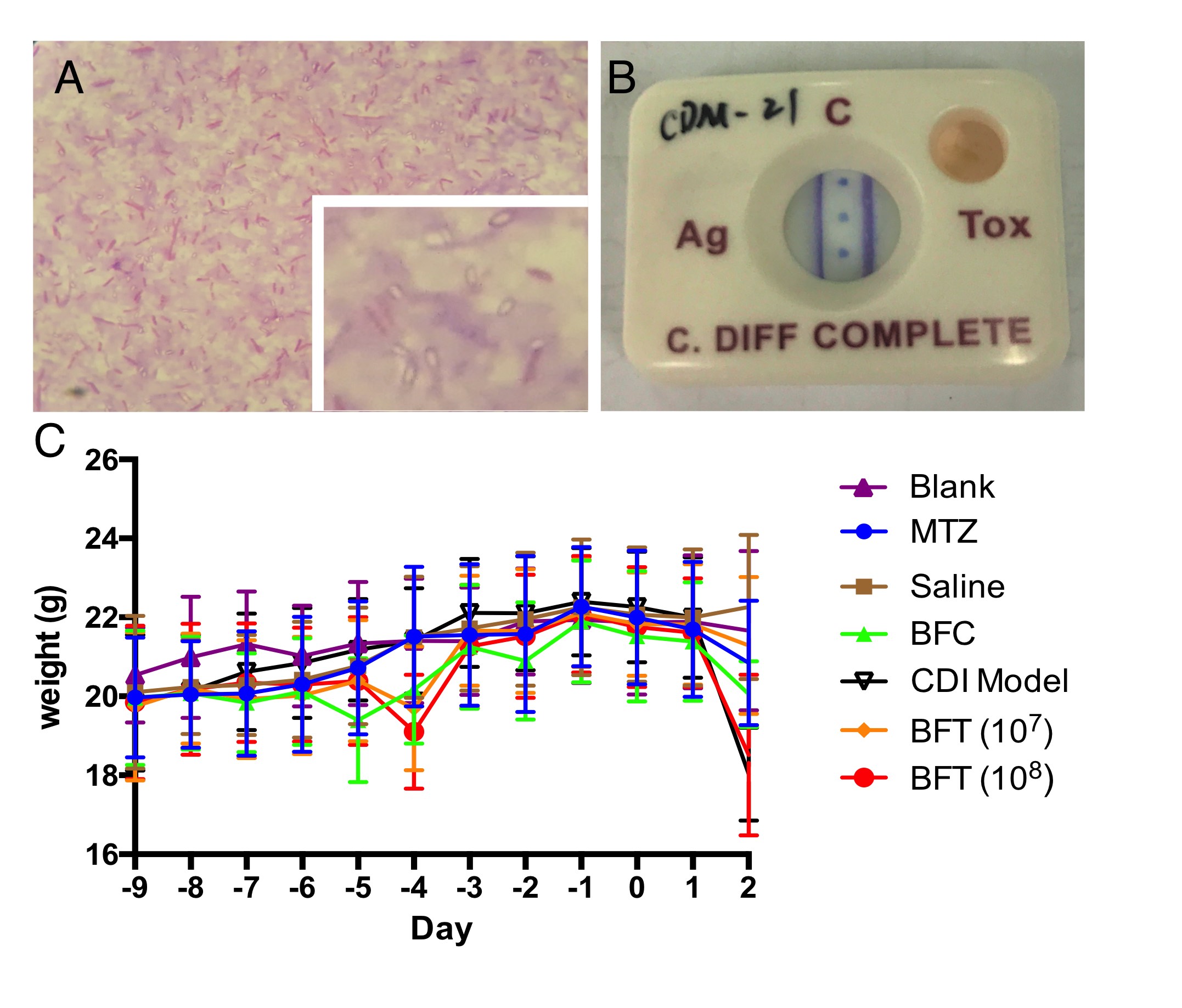

Supplement: Supplementary file 2 [file Image_1.JPEG]

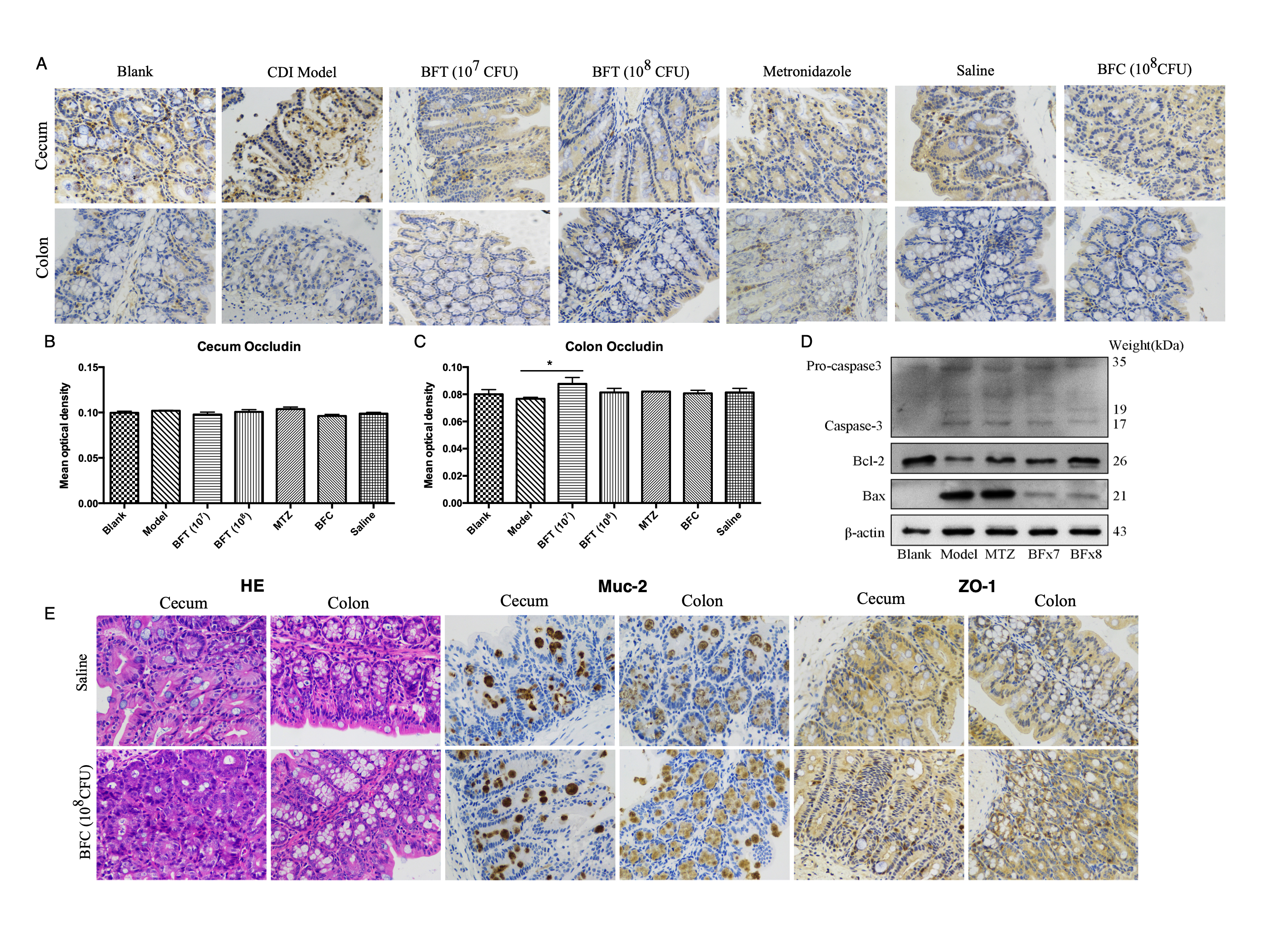

Supplement: Supplementary file 3 [file Image_2.JPEG]

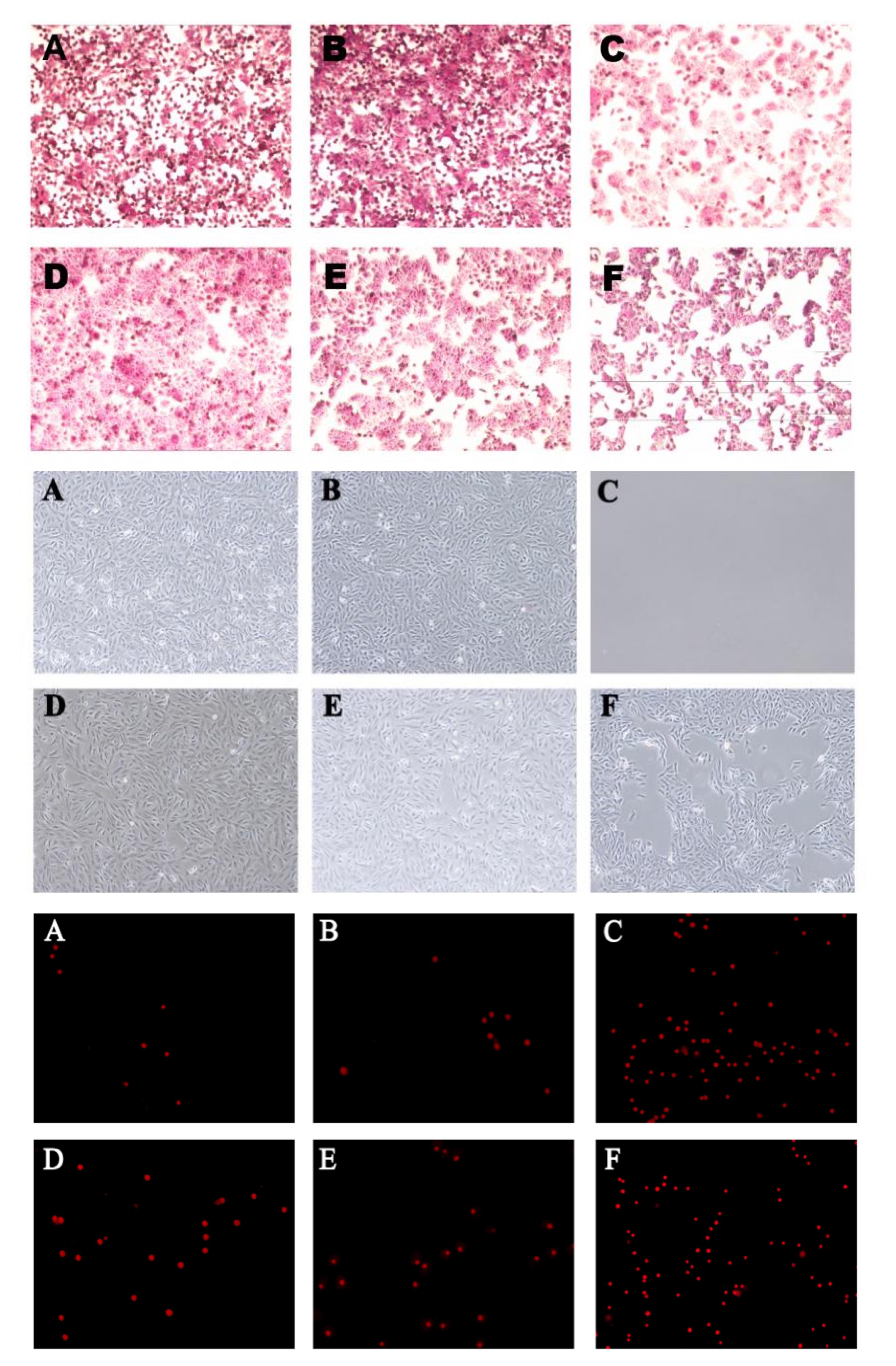

Supplement: Supplementary file 4 [file Image_3.jpeg]

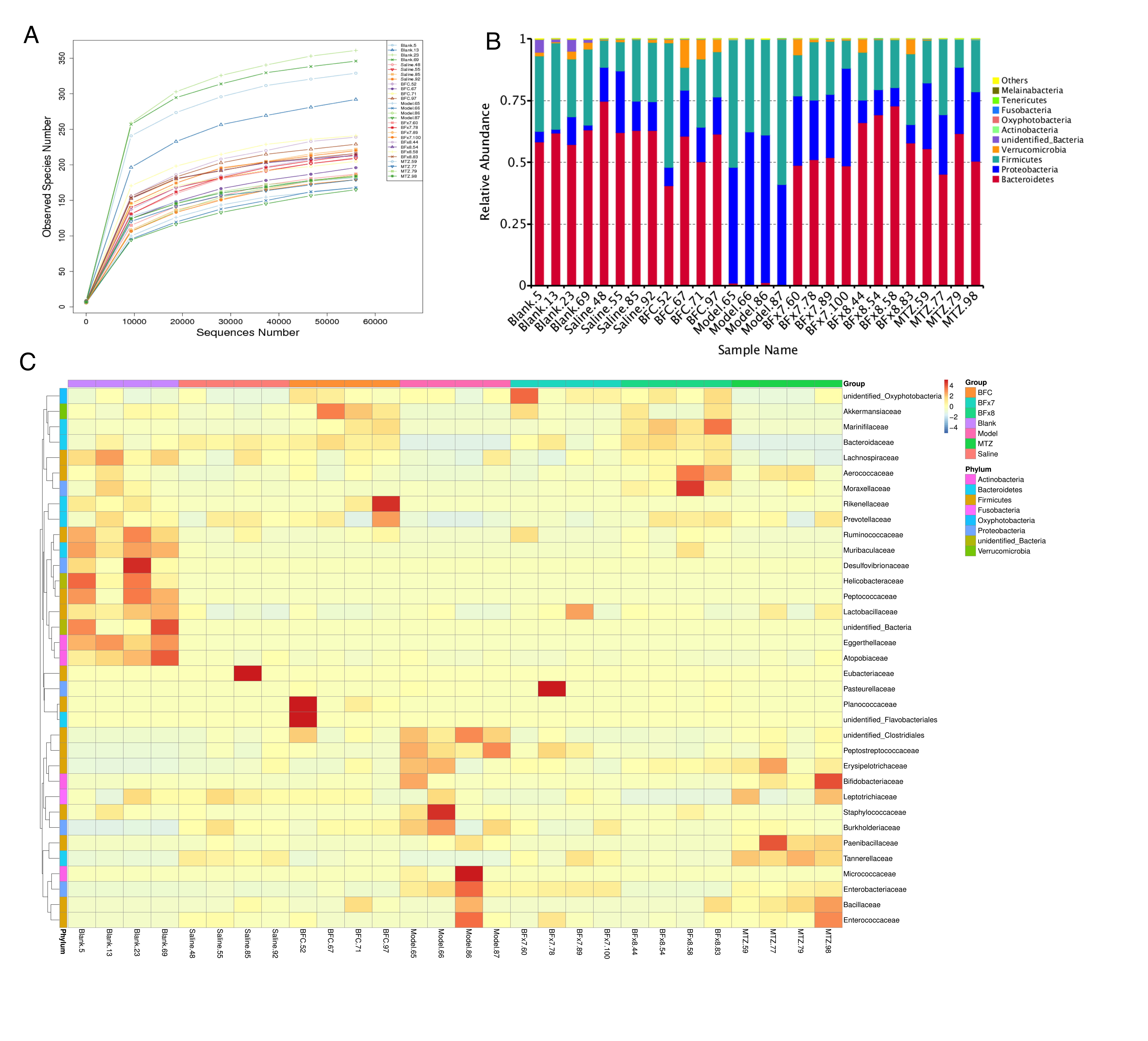

Supplement: Supplementary file 5 [file Image_4.JPEG]

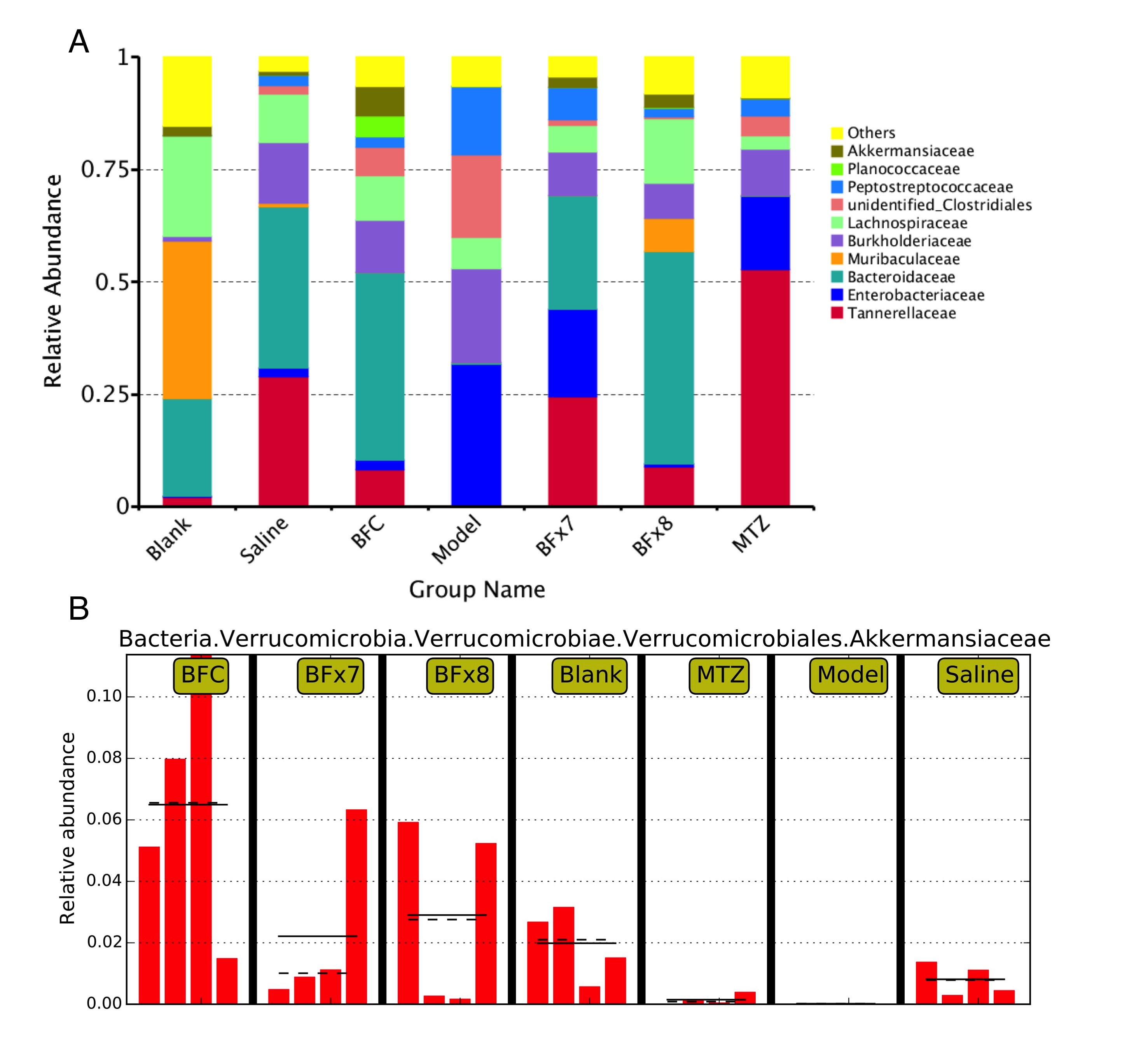

Supplement: Supplementary file 6 [file Image_5.JPEG]
